# Oral microbial taxa associated with risk for SARS-CoV-2 infection

**DOI:** 10.3389/froh.2022.886341

**Published:** 2022-09-02

**Authors:** Nicholas Callahan, Meryana Hattar, Thawab Barbour, Guy R. Adami, Nadia Kawar

**Affiliations:** ^1^Department of Oral and Maxillofacial Surgery, College of Dentistry, University of Illinois at Chicago, Chicago, IL, United States; ^2^Department of Oral Medicine and Diagnostics, College of Dentistry, University of Illinois at Chicago, Chicago, IL, United States; ^3^Department of Periodontics, College of Dentistry, University of Illinois at Chicago, Chicago, IL, United States

**Keywords:** bacteria, COVID-19 risk, *Neisseria elongata*, 16S rRNA (16S rDNA), oral microbiota

## Abstract

**Hypothesis and objective:**

The oral and digestive tract microbial ecosystem has sparked interest because of its impact on various systemic diseases and conditions. The oral cavity serves not only as a reservoir for many potentially virulent microbiota but also as an important entry point and portal to the human body system. This is especially significant in the transmissibility of the virulent current pandemic virus SARS-CoV-2. The oral and digestive microbiome influences the inflammatory burden and effectiveness of the immune system and serves as a marker of activity of these host processes. The host immune response plays a role in infection susceptibility, including SARS-CoV-2. The purpose of this study is to investigate the role of specific salivary oral microbiome in susceptibility to SARS-CoV-2 infection.

**Methods and results:**

One hundred six subjects of known medical and dental history who consented to provide saliva samples between January 2017 and December 2019 were included in this study. Sixteen had become COVID-19 positive based on the PCR test by 3/01/2021. A comparison of oral microbiome bacteria taxa profiles based on 16S rRNA sequencing revealed differences between the two groups in this pilot study.

**Conclusions:**

These bacteria taxa may be markers of increased susceptibility to SARS-CoV-2 infection in the unvaccinated population.

## Introduction

The microbiome of the oral cavity and the digestive tract exists in a delicate homeostasis, and this balance of bacteria plays an important role in the body's immune and inflammatory potency levels [1, 2]. The oral and nasal mucosal membranes are the first entry point for many respiratory viruses, and the microbiome plays a significant role in the pathogenesis of these viral infections [3]. There is certainly precedent for the idea that microbiota, whether digestive (gut/oral) or respiratory, could play a role in the resistance and response to infection by viruses such as the SARS-CoV-2 virus [4], associated with 5.3 million deaths and 272 million cases worldwide by the end of 2021. Salivary microbiota, specifically bacteria, play a significant role in facilitating attachment of incoming microbes into its ecosystem and, from there, breakthrough to the mucosal membranes of the host system. Salivary microbiota have the potential to control viral uptake into cells and whether virus introduction into the host is sufficient for a productive, detectable infection. In addition, these microbiota can create molecules that alter host epithelial barriers [5].

Many studies have identified changes in the gut, oral, and nasopharyngeal bacteria that occur during and post SARS-CoV-2 infection vs. healthy controls [6–11]; however, the mechanism of these changes remains unknown. The SARS-CoV-2 virus has a profound effect on the oral cavity's function, with symptoms including loss of taste, dry mouth, dysgeusia, and opportunist infections that may alter oral microbiota [12]. We can presume that changes in other oral microbiota due to SARS-CoV-2 infection may contribute to these symptoms within the oral cavity. Additional work has attempted to study the changes in oral/gut bacteria that occur during the course of SARS-CoV-2 active infection and its correlation with severity of disease [8, 11, 13–15]. These studies shed light on bacteria that have the potential to increase the morbidity rate with SARS-CoV-2 infection at sites such as the oro-pharynx [16, 17].

The COVID-19 etiological agent, SARS-CoV-2 betacoronavirus, has high spreading capability, mediated by several vehicles, such as oral droplets [18–20]. The oral cavity might be considered a preferred route for SARS-CoV-2 entry, and there is ample evidence that spread primarily *via* a droplet [2]. These droplets can enter the human body through the mucosal membranes of, primarily, the nasal cavity *via* the ACE2 + TMPRSS2 + nasal epithelial cells [3]. While there has been more investigation of the nasal-lung axis of infection [4], infection can occur *via* the oral cavity [1, 2]. This is supported by high expression of both the ACE2 and TMPRSS2 receptors in the salivary glands and the oral cavity mucosa. While oral manifestation of SARS-CoV-2 infection occurs in oral tissues, it is not clear how often it is the primary site of SARS-CoV-2 initial infection [21]. After entry into the oral cavity, a successful infection requires survival of the virus in the saliva or on the mucosa until its adherence to the mucosal surface, followed by penetrance through the ECM binding to the ACE receptor for uptake, and then successful viral reproduction to the point that the level of viral particles exceed the threshold for detection. The question addressed here is whether certain salivary oral microbiota may facilitate attachment and penetration, increasing the risk of a successful infection process.

This was a retrospective study; the purpose was to investigate preexisting oral salivary bacterial diversity and its impact on susceptibility to SARS2-CoV-2 infection. Salivary samples were collected from patients of record, with known pre-pandemic medical and dental history, who were followed through the pandemic. The saliva microbiome is known to be relatively stable over time for individual adults [22, 23], and this method offered the unusual proposition that the salivary microbiome was known prior to infection. This allowed the inquiry into whether there are salivary bacteria taxa profiles associated with susceptibility to successful COVD-19 infections. Based on the location and time of the study in central Illinois, this analysis is of the association between successful infection by alpha, beta, and gamma variants of SARS-CoV-2 and levels of specific oral bacteria taxa (https://www.cdc.gov/coronavirus/2019-ncov/variants/variant-classifications.html).

## Materials and methods

### Study population and patient characteristics

This study was on a subset of subjects from a cross-sectional study conducted at the University of Illinois at Chicago College of Dentistry, General Practice and Prosthodontic clinics between 01/08/2017 and 06/21/2019 [24]. Approximately, 272 subjects provided written informed consent to participate in accordance with guidelines of the institutional ethics committee of the University of Illinois at Chicago, Institutional Review Board 1, which approved this study #2016-0696. This study was done in full accordance with the principles of the Declaration of Helsinki. Inclusion criteria: 18 years of age and older, available medical and dental record including a current medication list, a full periodontal exam, a visual, tactile, and radiographic caries exam, dentate or edentulous, and consent to supply a saliva sample. Exclusion criteria: presence of restored dental implants, removable partial dentures, maxillofacial defects scaling of teeth within the past 3 months; acute disease that requires urgent care, less than twenty natural teeth for the dentate subjects, antibiotic use within the past month or mouthwash in the last 12 h, vaccination of SARS-CoV-2 prior to the March 1, 2021 cutoff date, unknown COVID-19 history.

### Sample collection

Stimulated saliva was collected from patients asked to chew paraffin over a 5-min period as part of an earlier study [25]. The subjects all had had full dental exams prior to the study.

### Evaluation of COVID-19 status

All the subjects were contacted by telephone at least three times between April and May of 2021 before exclusion due to no response. Those who responded were subjected to a scripted telephone interview to determine presence of respiratory illness between February 1, 2020 and March 1, 2021, whether they had tested positive for COVID-19 by the PCR test, and whether they had been vaccinated for SARS-CoV-2 before March 1, 2021.

### Characterization of microbial community structure

Genomic DNA was extracted from saliva samples, followed by amplification of the V1–V3 variable region of bacterial 16S ribosomal RNA (rRNA) genes using the primer pair 27F/534R and a two-stage targeted amplicon sequencing as described previously [25, 26]. The second PCR amplification used FluidigmAccess Array barcoded primers to allow unique labeling of each sample at the University of Illinois at Chicago Sequencing Core, which was followed by sequencing on an Illumina MiSeq with the V3 kit to allow 600 cycles total.

For taxa assignment and measurement, reverse sequence reads from the FASTQ files were analyzed using the software package QIIME2 (v2022.2) [27]. Sequences were trimmed if the average quality was lower than 25. As a result, the read sequences were truncated at 262nt. DADA2-plugin in QIIME2 was used to denoise the sequence and generate feature data and feature tables for the dataset of DNA sequences [28]. Taxonomy assignment was done by classify-consensus-blast function using the Blast+ consensus taxonomy classifier to determine 98% match identity of the query sequences to the Human Oral Microbiome Database (v15.22) [29]. Of the 106 samples, there were 25,136 reads per sample on average (range, 12,645 – 36,906). There were 2,664,478 million reads total.

### Statistical analysis

Alpha diversity and Beta diversity analyses were performed using MicrobiomeAnalyst [30]. This analysis included calculation of the Shannon's Diversity Index of both species number and their distribution, and Chao1 indices of richness. These were compared between groups using a *t*-test within MicrobiomeAnalyst. Beta diversity analysis was visualized using the Bray–Curtis dissimilarity (non-phylogenetic) metric. MaAsLin2, using a Zero-inflated negative binomial regression with CSS normalization, allowed determination of taxa that were differentially represented in saliva from the subjects who became COVID positive vs. those who did not [31, 32]. This was done after internal batch correction by setting random_effects in the program to one of the two sequence runs. At least 2 reads were required per taxa measurement, and taxa that appeared in less that 10% of the total number of the samples were omitted. For statistical analysis of the study population demographics, two-tailed Fisher's Exact and Student's *T*-tests were run within the Kalediagraph program (Synergy Software). For analyses using MaAsLin2, all q values are determined using the Benjamini Hochberg procedure.

## Results

### Participants and population description

Approximately, 272 subjects who had provided saliva samples between November 2017 and December 2019 were contacted by phone during April 2021. Of these, 166 either did not know their COVID-19 status or were not reachable by phone and were excluded from the study. One hundred six subjects knew their prior COVID-19 infection status at the cut-off date of March 1, 2021; 16 had been PCR-verified COVID-19 positive at some point and were classified as positive. There were 90 subjects in the COVID-19 negative group. These people reported no respiratory illness between 1 February 2020 and 1 March 2021 and no positive SARS-CoV-2 PCR test at any point.

The salivary microbiome was compared between the positive and negative groups in this pilot study. Table 1 shows the general characteristics of the study group at the time of sample acquisition in terms of dental health, tobacco usage, number of prescribed medications, and age. The groups are similar overall, with a trend toward a higher level of tobacco usage in the COVID-19 positive group.

**Table 1:** Demographics of the study population.

**Demographics**		**COVID-19 negative**	**COVID-19 positive**	***p* value^a^**
Gender	Male	36	7	0.493
	Female	54	9	
Tobacco Use	Yes	14	5	0.126
	No	76	11	
Periodontal Disease	Yes	40	5	0.604
	No	50	10	
Dentate	Yes	80	14	0.312
	No	10	2	
Age	Mean	52.3 ± 15.7	47.2 ± 13.3	0.23
Medication Count	Mean	3.27 ± 4.62	1.73 ± 3.17	0.22
Caries	Mean	4.50 ± 7.88	4.13 ± 6.45	0.86

^a^Significance for gender, tobacco use, periodontal disease, and dentate status determined by the Fisher's exact test. Significance for age, medication count, and caries determined by the Student's T-test.

### Microbial diversity analysis and differentially abundant taxa

**Figure 1:**
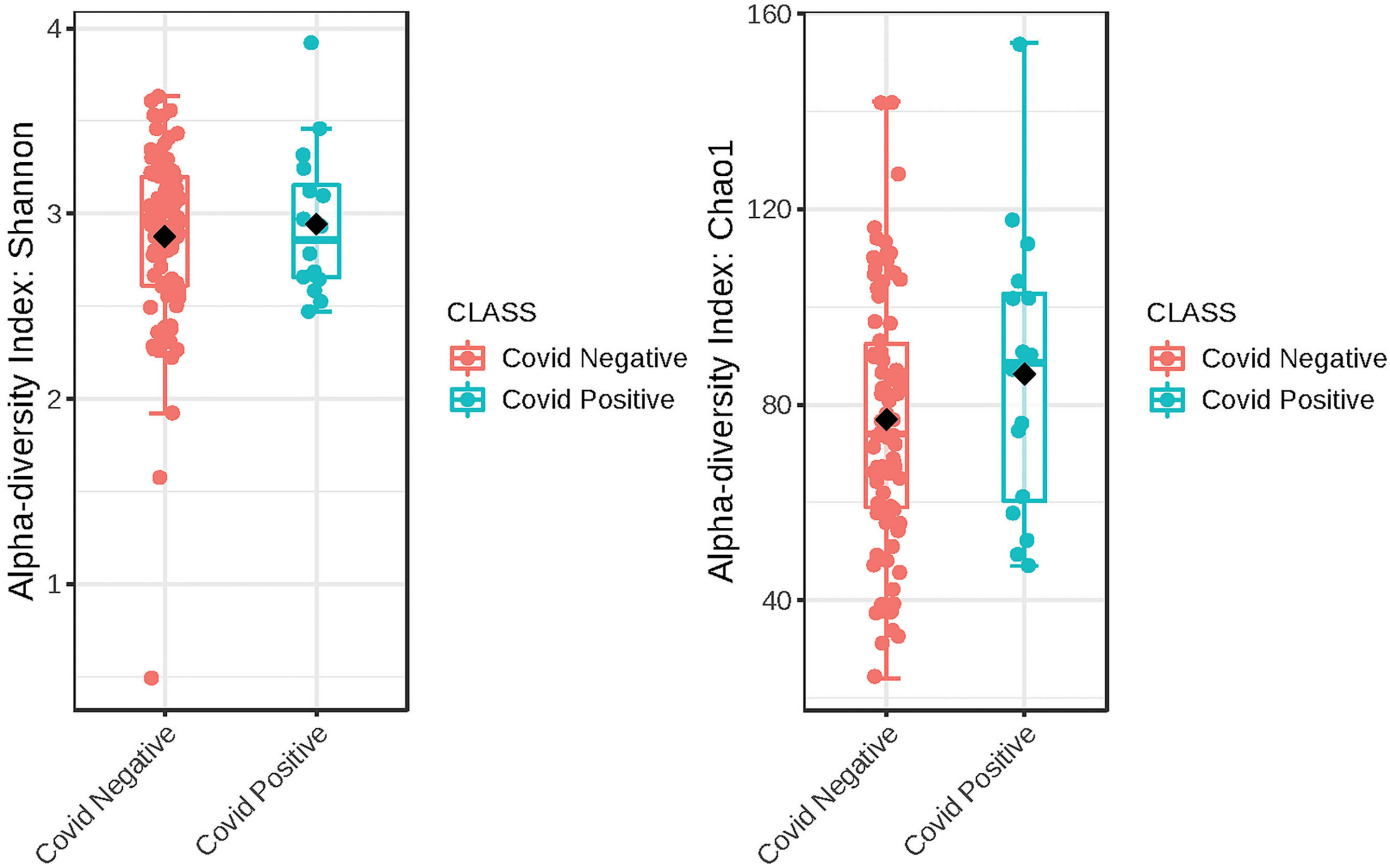
Phylogenetic diversity of saliva from subjects that self-report as being COVID-19 positive and those that did not. A box plot of Comparison of Shannon Diversity indices shows insignificant differences in taxa richness in the two groups, *p* < 0.543 and [*t*-test] statistic: −0.616. Comparison of taxa richness based on the CHAO1 indices reveals the same, *p* < 0.2510, (*t*-test) statistic: −1.19.

**Figure 2:**
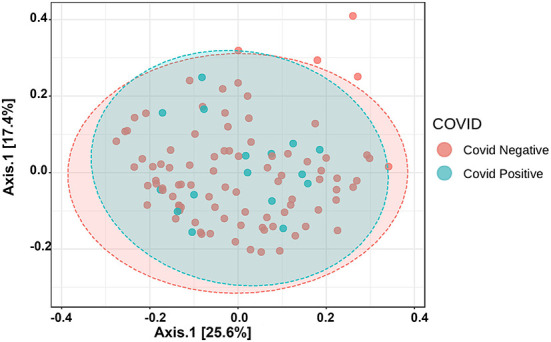
Principal coordinate analysis (PCoA) of saliva microbiome profiles of both groups based on Bray-Curtis Distances.

The Alpha diversity of the two groups shows no difference between the groups (Figure 1). The Beta diversity overall does not show a significant difference between the two groups (Figure 2). This is typical for saliva samples, which are a mixture of taxa from multiple niches.

**Figure 3:**
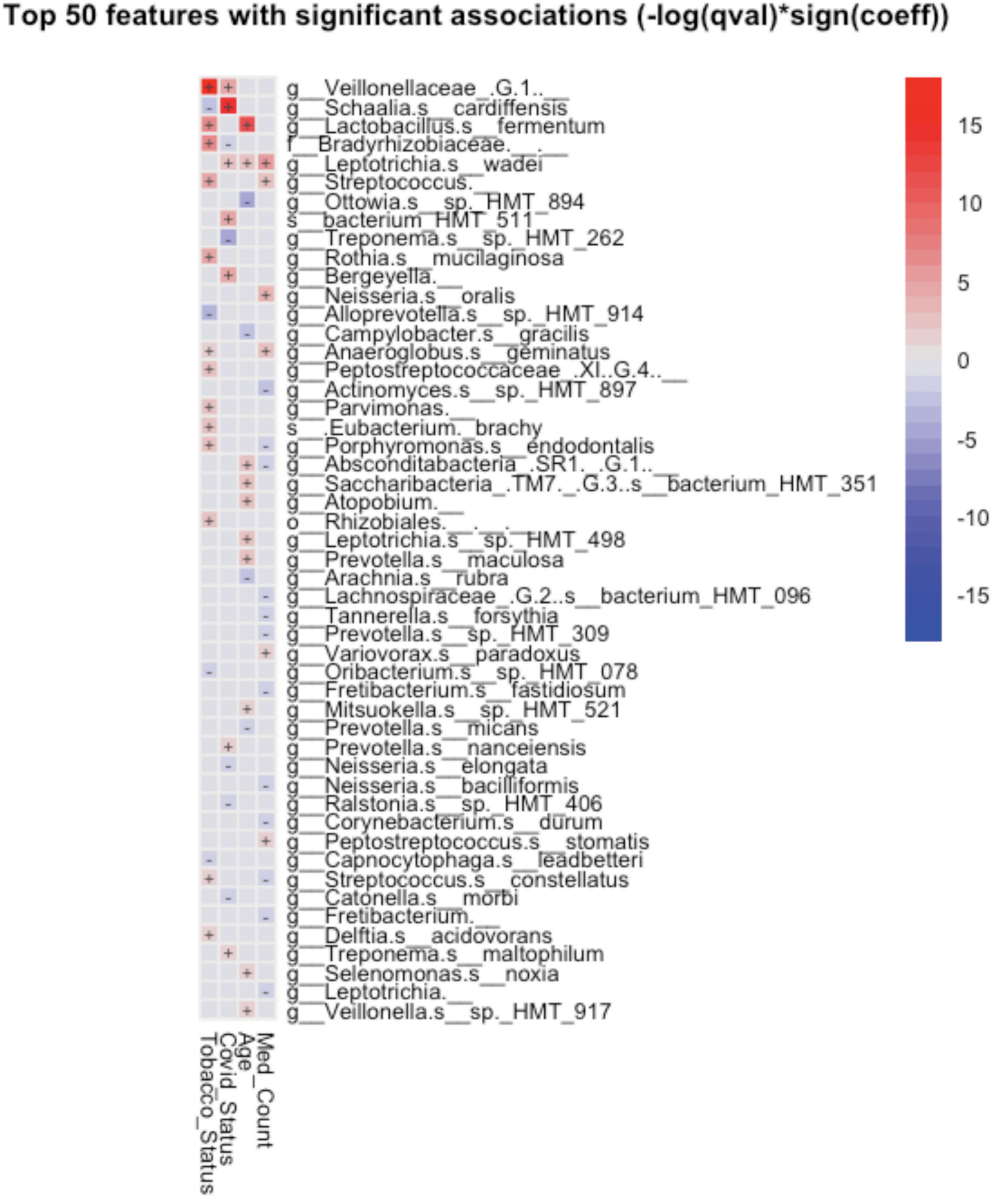
Significant associations (FDR < 0.25) between bacterial taxa and clinical variables detected by MaAsLin2-based multiple variable analysis. Each association such as future COVID-19 status and salivary taxa were adjusted for the remaining factors, such as subject's tobacco usage, age, and the number of medications prescribed to the subject.

Taxonomic analysis reveals limited differences between the two groups. Usage of analysis tool MaAsLin2 in Table 2 shows bacterial *taxa: Schaaliacardiffensis, g_Bergeyella, g_Bacteroidetes_[G-5] s__bacterium_HMT_511, g__Neisseria. s__elongata, g__Prevotella.s__dentalis*, which differ in levels between the two groups at FDR <0.1 (Table 2) [31, 32]. Multivariable analysis with correction for levels of tobacco usage, age, and medication count is shown in Figure 3; Supplementary Table 1.

**Table 2:** MaAsLin2-based determination of taxa that are differentially abundant in saliva of the subjects that became COVID-19 positive.

**Taxa**	**coef**	**stderr**	**Pval**	**qval^a^**	**COVID-19 +**
*g__Bacteroidetes_.G.5..s__bacterium_HMT_511*	1.59E+00	3.89E-01	4.27E-05	7.04E-03	Higher
*g__Schaalia.s__cardiffensis*	1.23E+00	3.21E-01	1.31E-04	1.08E-02	Higher
*g__Neisseria.s__elongata*	−1.05E+00	2.96E-01	3.69E-04	2.03E-02	Lower
*g__Bergeyella.__*	9.38E-01	2.78E-01	7.52E-04	3.10E-02	Higher
*g__Prevotella.s__dentalis*	1.52E+00	4.79E-01	1.52E-03	5.03E-02	Higher

^a^Q or FDR values below.1 (1.00E-01) are considered significant. The coef refers to the model coefficient value or effect size, the stderr is the standard error of the model, while the qval is the corrected significance using the Benjamini Hochberg FDR.

## Discussion

A small group of oral taxa was shown to be differentially abundant in subjects who went on to be COVID-19 positive. Two of these, *Prevotella* and *Neisseria*, are of interest in that they have been tied to COVID-19 in the past. Multivariable analysis (Figure 3) revealed these taxa may be associated in part with differences in age, tobacco usage, and/or numbers of medications required in the groups studied.

A species of the genus *Prevotella* was observed to be more abundant in the saliva of patients who went on to become SARS-CoV-2 positive (Table 2).

Earlier studies have noted *Prevotella pallens* was enriched in saliva samples from patients with COVID-19 with active disease, while *Rothia mucilaginosa* and some *Streptoccoci* species were at lower levels [13]. A second study of patients with active disease vs. healthy controls noted oral mucosa were enriched for *Haemophilus parainfluenzae, Veillonella infantium, Soonwooa purpurea, Prevotella salivae, Prevotella jejuni*, and *Capnocytophaga gingivalis*, while displaying lower levels of a number of taxa [7]. A third study did not see this higher level of *Prevotella* at least on the genus level in saliva samples of patients with COVID-19 [33]. Based on the enrichment of *Prevotella* genes in lung samples from COVID-19 patients with active disease and computational identification of possible roles for *Prevotella*-encoded proteins in host-viral interactions, there is speculation that this enrichment of *Prevotella* in some patients could play a fairly direct role in stimulating SARS-CoV2 infection and subsequent disease [34]. Comparison between studies is made difficult by the variety of 16S rRNA amplicons that are examined in the different studies. Nevertheless, the higher level of the single *Prevotella* species seen in the subjects who became COVID-19 positive (Figure 3; Table 2) may provide some support for that model.

Low levels of the bacteria genus *Neisseria* in patients with COVID-19 have been proposed to play a role in increased inflammation in some patients [35]. There are several reports of lower levels of *Nesseria* overall in patients with COVID-19, and Iebba et al. report lower levels, specifically of *Neiserria elongata* in oral rinses from patients with COVID-19 at FDR <0.11 [7, 8, 15]. As shown in Table 2, *Neiserria elongata* was at a lower level in the patients who went on to contract COVID-19. One might speculate that this species is lower in COVID-19-positive patients before they get the disease.

While the taxa that differ between the two groups may serve as markers of COVID-19 disease susceptibility, the mechanism is obscure. Differences in resistance to viral establishment could be tied to genetics of the host, inflammatory state, immune function, and the microbial organisms that can affect each other [36]. At the time of the initiation of this work, it was not clear if the oral cavity is a primary site of SARS-CoV-2 infection, and this is still unclear [37, 38]. For that reason, the few differences seen in salivary microbiome in the group that had been COVID-19 positive could be manifestations of lifestyle associated with increased risk anywhere in the body. This is not an unprecedented concept, in that there is ample evidence of oral microbiome differences being linked to differential disease risk throughout the body [39].

This study has several limitations: it was an observational, retrospective, single-center study. Multiple factors can cause minor differences in oral microbiota, so any relationships may be indirect, and the number of COVID-positive subjects limits sensitivity of the study [39, 40]. In addition, it is possible that a small number of COVID-negative subjects had been positive at some time, but this was not known to them [41]. However, the large number of the subjects in the COVID-negative group would minimize that as a source of error. We used V2-V3 16S rRNA analysis, which provides reproducible information on the levels of a large percentage of known oral bacteria taxa, but not all oral bacteria were measured [42]. This study has additional limitations: (1) A lack of functional analysis of microbial genes, (2) Collection of saliva in some cases had occurred over a year prior to the COVID-19 pandemic, (3) The date of COVID-19 positivity was not recorded. (4) The presence of restorations such as dental crowns was not considered, although the patients with implants were excluded. A prospective study with more frequent saliva collection would have addressed some of these limitations.

Given the March 1, 2021 cutoff for previous infection status and the location in the United States, findings may be limited to the alpha and beta SARS2-CoV-2 strains [43]. In addition, salivary properties of viscosity, pH level, and immunoglobulin and inflammatory cytokines levels were not assessed, which may play a role in the protective mechanism against microbial attachment and penetration [44].

## Conclusion

These bacteria taxa present in the saliva may be associated with increased risk of early SARS-CoV-2 infection in the unvaccinated population. This study can serve as a prelude to the study of the diverse oral microbiota and its effect on the protective mechanism or facilitation of infection by respiratory viruses.

## Data availability statement

The datasets presented in this study can be found in online repositories. The names of the repository/repositories and accession number(s) can be found below: BIoproject accession numbers: PRJNA674379 and PRJNA739492.

## Ethics statement

The studies involving human participants were reviewed and approved by UIC IRB. The patients/participants provided their written informed consent to participate in this study.

## Author contributions

NC, MH, TB, GA, and NK contributed to the performed data collection and analysis. NC and GA wrote the first draft of the manuscript. All the authors contributed to the editing and agree with the content of the manuscript.

## Conflict of interest

The authors declare that the research was conducted in the absence of any commercial or financial relationships that could be construed as a potential conflict of interest.

## Publisher's note

All claims expressed in this article are solely those of the authors and do not necessarily represent those of their affiliated organizations, or those of the publisher, the editors and the reviewers. Any product that may be evaluated in this article, or claim that may be made by its manufacturer, is not guaranteed or endorsed by the publisher.
